# Effect of interdisciplinary obesity care on metabolic markers and body weight in people with type 2 diabetes in a rural setting: A randomised controlled trial

**DOI:** 10.1111/cob.12715

**Published:** 2024-11-07

**Authors:** Giuliana O. Murfet, Iain K. Robertson, Sharon P. Luccisano, Michelle L. Kilpatrick

**Affiliations:** ^1^ School of Public Health University of Technology Sydney Ultimo New South Wales Australia; ^2^ Diabetes Centre Tasmanian Health Service Burnie Tasmania Australia; ^3^ School of Health Sciences University of Tasmania, Newnham Campus Launceston Tasmania Australia; ^4^ Menzies Institute of Medical Research University of Tasmania, Medical Science Precinct Hobart Tasmania Australia

**Keywords:** diabesity, diabetes, interdisciplinary management, nurse practitioner, obesity policy, obesity, rural health

## Abstract

Management of type 2 diabetes includes medications that can unintentionally increase obesity and insulin resistance. This unblinded, single‐centre, randomised controlled trial focused on rural Australian adults with type 2 diabetes (aged 18–75 and body mass index [BMI] >30 kg/m^2^), measuring the effectiveness of a tailored interdisciplinary obesity care approach compared with usual diabetes care. Led by a nurse practitioner with allied health support (dietitian ± psychologist and physiotherapist), the bariatric treatment involved reducing weight‐gaining medications, a 500‐calories/day deficit, an unsupervised exercise program emphasising movement/strength and psychotherapy, 3‐monthly to 24‐months with support phone calls at weeks 2, 4, 8 and 10. Outcomes from the 224 (113 intervention/111 control) participants were differences in biomedical and physical markers within‐ and between‐groups estimated using multivariate mixed‐effects linear regression between recruitment and 6‐monthly follow‐ups. Greater change occurred in intervention compared with control groups at 12 and 24 months in mean: body weight (−5.9 kg [95% confidence interval, CI: −8.53, −3.23] and −9.0 kg [95% CI: −13.2, −4.77]); BMI (−2.03 kg/m^2^ [95% CI: −2.92, −1.15] and −3.51 kg/m^2^ [95% CI: −4.93, −2.08]); and glycated haemoglobin (−0.26% [95% CI: −0.69%, 0.18%] and −0.63% [95% CI: −1.17%, 0.08%]). The control group showed a significant increase in mean leptin level, resulting in a between‐group difference (−27.1 [95% CI: −42.7, −11.5]). Lipids and blood pressure differences were inconclusive, while exploratory analysis showed greater decline in estimated glomerular filtration rate in the control group. The interdisciplinary obesity approach, compared with usual diabetes care, resulted in sustained weight loss and improved diabetes control over 2 years. *Trial registration number*: ACTRN12622000240741.

**What is already known about this subject**

- *Obesity* is a complex condition that is increasing in prevalence, particularly in rural, regional settings.
- The complexity increases for people with type 2 diabetes, and diabetes medicines or medications used to manage comorbidities can make it difficult to lose weight.

**What this study adds**

- The study provides evidence to support interdisciplinary management of obesity in people with type 2 diabetes that contribute to positive health outcomes, including weight loss and improvement in diabetes‐related metabolic markers.
- The results can guide policy for obesity services in rural, regional areas.

## 1 INTRODUCTION

Obesity is a common, chronic and complex condition with genetic, environmental, physiological and behavioural determinants.^1^, ^2^, ^3^, ^4^ Obesity causes significant comorbidities, particularly type 2 diabetes, cardiovascular disease, obstructive sleep apnoea, non‐alcoholic steatohepatitis, osteoarthritis, and prostate, breast and colon cancers.^3^, ^4^, ^5^ For people living with obesity and type 2 diabetes, the risks of diabetes‐related complications, adverse cardiovascular outcomes and cancer are increased.^1^, ^3^, ^4^

Obesity and diabetes share pathophysiological links, which should be considered concurrently during clinical management.^6^ For example, appetite regulation is complex and influenced by mediators from adipose tissue, the pancreas and the gut.^4^, ^7^ Excess adiposity promotes inflammatory mediators causing hyperglycaemia that weakens hunger‐inhibiting signalling, which reduces body weight regulation.^4^, ^8^, ^9^, ^10^, ^11^ These are all intensified in people with type 2 diabetes because of insulin resistance and when managed with weight‐gaining glucose‐lowering medicines.^3^, ^4^, ^8^, ^9^, ^10^ However, weight reduction has improved insulin sensitivity, endothelial function, inflammatory markers and coagulation.^4^, ^8^, ^9^, ^10^, ^11^, ^12^

Obesity and diabetes benefit from lifestyle management, such as behavioural changes in nutrition intake and physical activity.^3^, ^4^, ^10^ When people live with both obesity and diabetes, clinical oversight of medication management can prevent complications and help detect and manage comorbidities to improve outcomes.^10^, ^13^ Simultaneously, multidisciplinary obesity support can lead to sustained weight loss, improved metabolic markers such as glycated haemoglobin (HbA1c) and lipids, and reduced cardiovascular risk.^3^, ^10^, ^13^, ^14^, ^15^ However, people in rural/remote areas often experience reduced access to health services, including allied health, and limited nutritious dietary and physical activity options and environments.^16^, ^17^ These factors challenge effective health behaviour changes in obesity and diabetes management.

Evidence of longer‐term effectiveness of diverse models of obesity care is needed, especially multidisciplinary models addressing geographical and financial barriers to accessing services.^14^, ^16^, ^17^, ^18^, ^19^ Recognising this, a rural population was selected as the target for this study to assess the effectiveness of a rural, interdisciplinary (multi‐discipline and team‐based) obesity model of care on weight loss and metabolic factors in adults living with obesity and type 2 diabetes. We hypothesised that participants with access to an individualised obesity program involving a dietitian, physiotherapist or exercise physiologist, psychologist and medical management from a diabetes nurse practitioner (NP) would exhibit improved weight and metabolic outcomes compared with usual diabetes care provided by a diabetes educator and endocrinologist.

## 2 METHODS

### 2.1 Study setting

The AMOS trial (2015–2019) took place in two Tasmanian rural/regional sites: North‐West Regional Hospital, Burnie, and Devonport Community Health Services Centre. The Tasmanian Health Service (North‐West) serves 125 000 people with higher rates of obesity, chronic conditions and socioeconomic disadvantage than the national average.^1^, ^20^ Diabetes prevalence ranges 5.3%–8.2% (mean 6.9%) in council areas. The Diabetes Centre delivers care to 8000 people living with type 2 diabetes, 89% with overweightness or obesity, with a mean body mass index (BMI) 44.5 kg/m^2^. The NP holds a PhD, Master of Nursing, Master of Science and Postgraduate Diploma of Diabetes Education. Both the NP and dietitian were credentialled diabetes educators (CDE).^21^

### 2.2 Participants

*Inclusions*: Existing Diabetes Centre patients aged 18–<75 years, BMI > 30 kg/m^2^ and type 2 diabetes. *Exclusions*: People diagnosed with cognitive impairment, intellectual disability, uncontrolled psychotic illness, pregnancy or breastfeeding.

### 2.3 Trial design

A pragmatic single‐centre, randomised, unblinded trial comparing outcomes between intervention (the Assessment and Management of Obesity and Self‐maintenance [AMOS] clinic) and control group (usual Diabetes Centre care) at recruitment, 6, 12, 18 and 24 months. The full study protocol is available on the Australian New Zealand Trial Registry website.^22^

#### 2.3.1 Randomisation

##### Sequence generation

**FIGURE 1:**
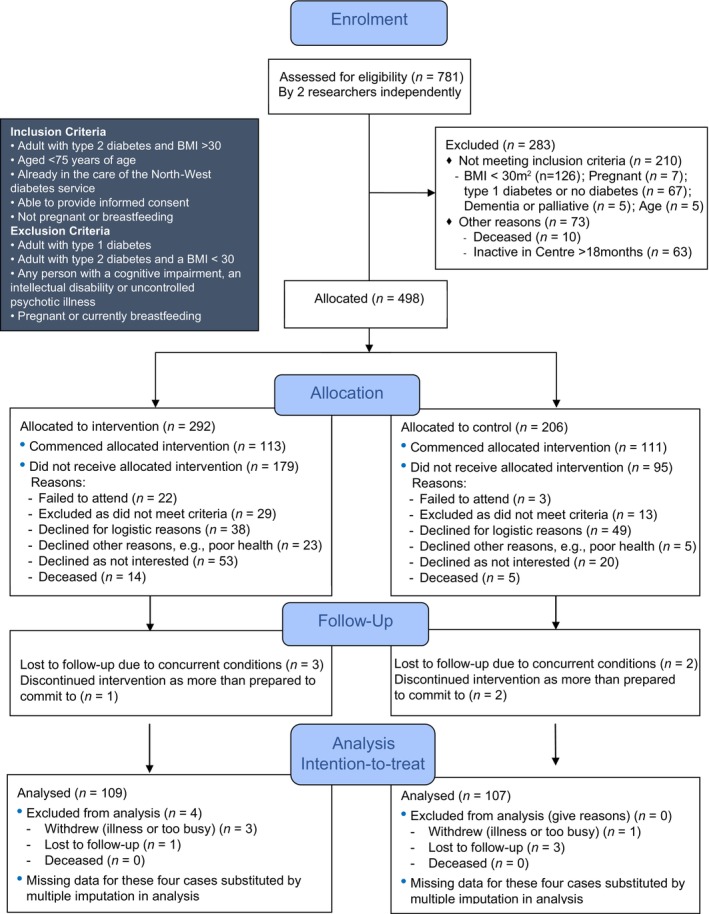
CONSORT flow diagram of the progress through the phases for participants in the randomised controlled trial. BMI, body mass index.

An independent researcher from the University of Melbourne used Microsoft Excel random number generator to develop the groups assignment list. *Allocation concealment*: Due to funding criteria requiring most funds to be spent on bariatric surgery, eligible participants were listed in order of BMI, then numbered, with one being the highest BMI. Thus, those with a higher BMI would be the first to be invited to the corresponding assigned group. *Implementation*: Two researchers identified the 781 eligible adults who had attended an endocrinologist appointment at the Diabetes Centre between January 2014 and May 2015 using the centre's database (see Figure 1). A Research Officer contacted potential participants by phone using a script to describe the trial. Interested participants provided written informed consent at the first consultation and were enrolled.

#### 2.3.2 Trial treatments

##### AMOS clinic

**FIGURE 2:**
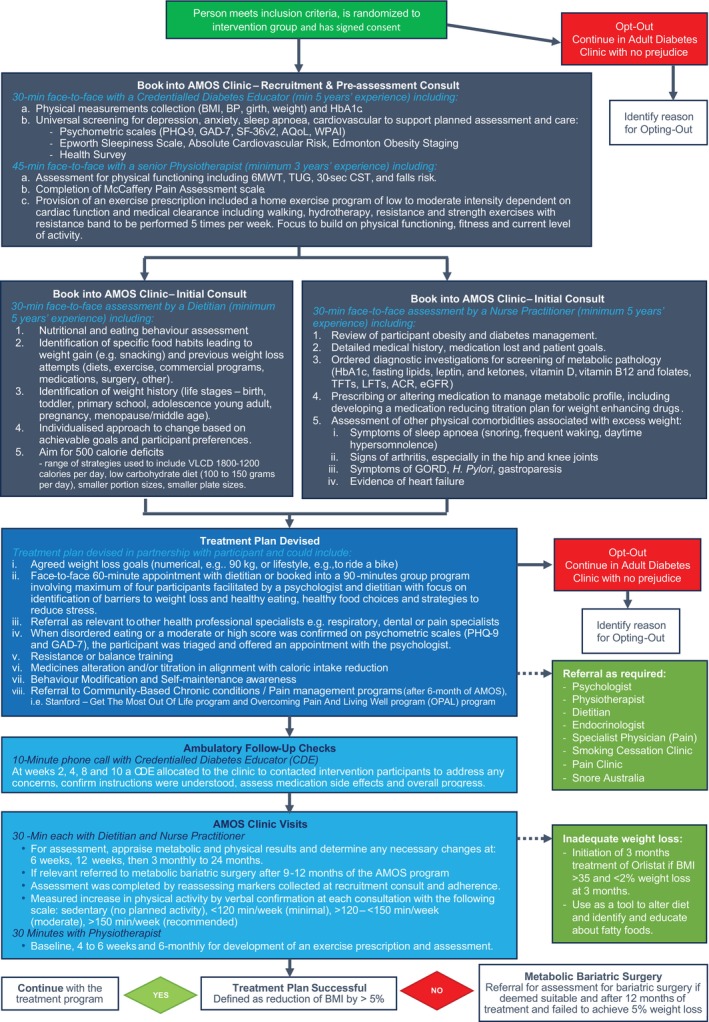
Intervention—AMOS Clinic (tailored obesity care with focus on self‐maintenance). BMI, body mass index; BP, blood pressure.

The intervention focus was to provide the person living with obesity and type 2 diabetes with individualised self‐maintenance skills to support weight loss and manage other health complications, including diabetes. Clinical strategies were the provision of NP‐led interdisciplinary obesity care, including medical assessment; allied health support (dietitian, physiotherapist, psychologist); reducing or substituting weight‐promoting for weight‐neutral/loss medicines (e.g. altering insulin to a glucagon‐like peptide‐1 [GLP‐1] analogue or reducing pregabalin doses); titrating diabetes medication regimens to facilitate weight loss without compromising diabetes control (i.e. adjusting treatment in alignment with caloric reductions to prevent hypoglycaemia); and bariatric surgery referral, if appropriate (20 surgeries funded) (see Figure 2).

An NP and dietitian delivered core AMOS Clinic consultations face‐to‐face at recruitment, 6 and 12 weeks, then 3‐monthly intervals until 24‐months. Participants saw the physiotherapist at recruitment, with further support available at 4 weeks, 6 and 12 months. In weeks 2, 4, 8 and 10, participants received support phone calls from a CDE to address questions, ensure they understood instructions and assess for medication side‐effects.

Participants were referred to a psychologist when initial screening showed moderate‐to‐severe anxiety or depression scores, or when a participant reported disordered eating. The intensity and length of psychological support were tailored to the needs of each client with up to six consultations, including treatment for trauma, depression or anxiety, stress management, psychosocial counselling, skills development (e.g. emotion regulation, anger management, assertiveness), health behaviour change support, bariatric surgery pre‐assessment and post‐surgery support. Treatment approaches drew from cognitive behavioural, exposure/response prevention, solution‐focused and interpersonal therapies.

In the trial, participants could engage at their preferred level and opt‐out of any intervention therapies. Participants were invited to a 1.5‐h AMOS Group session between weeks 4 and 12, where a dietitian and psychologist presented basic healthy eating and stress management advice and offered the *Get The Most Out Of Life* and *Overcoming Pain and Living Life* (OPALL) (if relevant) programs at the 6 months point.^23^, ^24^ These local primary care programs establish self‐management strategies for managing chronic conditions and pain.

##### Usual diabetes care

The comparator was usual Diabetes Centre care provided to participants attending the Diabetes Centre; an initial assessment and health screening by a CDE every 3 to 6 months, including diabetes self‐management education and insulin titration 1–4 weekly when relevant via ambulatory care. Additionally, an endocrinologist assessment 6‐ or 12‐monthly focused on diabetes management and comprised referral to a dietitian when HbA_1c_ >9% (75 mmol/mol).

### 2.4 Trial outcomes

The primary outcomes were changes in body weight and HbA_1c_, with reductions of −5% and −0.5%, respectively, deemed clinically relevant treatment effects,^3^, ^4^, ^8^, ^10^, ^12^, ^14^, ^25^ at recruitment, 6, 12, 18 and 24 months. Secondary outcomes were changes in fasting total, high‐density (HDL) and low‐density (LDL) lipoprotein cholesterol and leptin, estimated glomerular filtration rate (eGFR), blood pressure (BP) and obesity staging at those times.

### 2.5 Data collection procedure

An accredited practising dietitian conducted interviews 3‐monthly to assess weight loss attempts and habitual food intake. Body weight and height were measured at AMOS Clinic appointments using digital Tanita scales, with no shoes and minimal clothing. BMI was calculated (weight {kg}/height {m}^2^). Resting BP was measured (Omron BP‐203RPEIII VP‐1000 device). Fasting blood lipids (mmol/L), leptin (ng/mL), ketones (mmol/L), eGFR (mL/min/1.73 m^2^) and urine albumin creatinine ratio (ACR) (mg/mmol) were analysed by a registered pathology service (North‐West Pathology Diagnostic Services Pty Ltd) at recruitment and 6‐monthly. HbA_1c_, reported as % and mmol/mol, were assessed using the Abbott Afinion‐2 Analyzer at recruitment and 3‐monthly.

Obesity grading was allocated in alignment with the World Health Organisation stages; Obesity I (BMI of ≥30–34.9 kg/m^2^ [obesity]), Obesity II (≥35–39.9 kg/m^2^ [severe obesity]) and Obesity III (≥40 kg/m^2^ [morbid obesity]). The Edmonton Obesity Staging System (EOSS), a five‐staged system of obesity classification considering the metabolic, physical and psychological parameters to determine the optimal obesity treatment, was calculated.^26^

### 2.6 Statistical analysis

The trial analysis estimated mean differences in outcome measures at 6‐monthly follow‐ups on an intention‐to‐treat basis using multivariate mixed‐effects linear regression. Primary and secondary outcomes including mean body weight, HbA_1c_, BMI, eGFR, total, HDL and LDL cholesterol, systolic and diastolic BP and leptin (standard deviation [SD]) and change (Δ; 95% confidence intervals [CI]; *p*‐values and CI were corrected for multiple comparisons by the Sidak method) were adjusted for age, gender, smoking and EOSS between visits, treating time as a random effect using an unstructured covariance matrix.

Sensitivity analyses were conducted to investigate primary outcomes for participants in each group categorised by bariatric surgery recipients and non‐recipients. The differences at each subsequent visit were corrected for the difference at the first visit. Missing values were imputed (50 imputations) using chained equations. Intention‐to‐treat analyses meant some participants in both groups received bariatric surgery during the intervention or follow‐up.

The sample size of 212 participants (106 to each group) was calculated to detect significant differences in one or more following outcome measures: HbA_1c_, EOSS status and absolute cardiovascular risk between intervention and control groups, assuming a Cohens d of 0.5, power of 0.95 and alpha of 0.05 based on literature estimates. All analyses were performed using Stata 16.1 MP2 (StataCorp LLC, College Station, Texas, USA).

## 3 RESULTS

### 3.1 Participants

Staggered recruitment began in February 2015 and completed in February 2017, with 113 intervention participants and 111 control participants. More participants attended the Devonport Community Health Services Centre (55% of total participants [*n* = 124/224]: 58% in intervention group, [*n* = 65/111]), than the North‐West Regional Hospital, Burnie (45% of total participants [*n* = 100/224]: 42% intervention [*n* = 48/111]), with care delivered by the same health professionals. Lost to follow‐up/withdrawn was similar at 12 months (*n* = 9 intervention vs. *n* = 10 control) and 24 months (*n* = 20 vs. *n* = 27, respectively), and deceased participant numbers were equal across the groups at 24 months (*n* = 4 vs. *n* = 4).

#### 3.1.1 Baseline characteristics

There were differences in several covariates between the groups; notably gender, glucose‐lowering medicines, EOSS and renal function with more intervention participants females and on more glucose‐lowering medications (see Table 1). Mean age, HbA_1c_, BMI, diabetes duration, BP, lipid profile, obesity staging categories and smoking rates were similar, as were proportions on lipid‐lowering medication and insulin between AMOS trial groups.

**TABLE 1:** Characteristics of participants at recruitment.

Initial characteristics	Intervention (*N* = 113)	Control (*N* = 111)	*p*‐Value^a^
Female sex, *n* (%)	68 (60%)	49 (44%)	.023^b^
Age (years), mean (SD: range)	57.5 (9.7: 33, 74)	58.2 (11.4: 24, 75)	.63^c^
Previous bariatric surgery, *n* (%)	7 (6.2%)	8 (7.2%)	.80^b^
Level of education, *n* (%)			.77^d^
Grade 6 (primary school)	10 (8.9%)	3 (2.7%)	
Grade 7	25 (22.1%)	36 (32.4%)	
Grade 10 (secondary school)	42 (37.2%)	37 (33.3%)	
Grade 11–12	9 (8.0%)	13 (11.7%)	
TAFE or trade certificate	16 (14.2%)	14 (12.6%)	
University degree	11 (9.7%)	8 (7.2%)	
Indigeneity, *n* (%)	13 (11.5%)	13 (11.7%)	1.00^b^
BMI, *M* (SD)	40.0 (7.1)	40.7 (8.7)	.46^c^
Edmonton obesity staging system, *n* (%)			.023^d^
Stage I	2 (2%)	0 (0%)	
Stage II	87 (77%)	74 (67%)	
Stage III	24 (21%)	37 (33%)	
Obesity staging, *n* (%)			.67^d^
Stage I (obese)	33 (29%)	32 (29%)	
Stage II (severe obesity)	35 (31%)	30 (27%)	
Stage III (morbid obesity)	45 (40%)	49 (44%)	
Diabetes duration (years), *M* (SD)	13.9 (9.1)	13.0 (8.4)	.44^c^
No. of medications overall, median (IQR)	8 (6, 11)	9 (6, 11)	.44^d^
No. of BP medication per person, median (IQR)	1 (1, 2)	2 (1, 3)	.10^d^
No. of lipid‐lowering medication per person, *n* (%)			.83^d^
0	27 (24%)	24 (22%)	
1	69 (61%)	71 (64%)	
2	16 (14%)	15 (13%)	
3	1 (1%)	1 (1%)	
No. of glucose‐lowering medicines not inc. insulin per person, *n* (%)			<.0001^d^
0	1 (1%)	5 (5%)	
1	23 (20%)	48 (43%)	
2	43 (38%)	39 (35%)	
3	41 (36%)	19 (17%)	
4	5 (4%)	0 (0%)	
No. of participants on insulin	68	71	.58^b^
Total daily dose of insulin, median (IQR)	86 (48, 128)	100 (64, 135)	.15^d^
Systolic BP, *M* (SD)	133 (16.0)	132 (20.8)	.69^c^
Diastolic BP, *M* (SD)	74 (9.6)	74 (9.7)	.85^c^
HbA_1c_, median (IQR)	8.0% (7.3%, 9.5%)	8.0% (7.0%, 9.2%)	.50^d^
Total cholesterol (mmoL/L), *M* (SD)	4.3 (1.0)	4.1 (1.2)	.31^c^
Low‐density lipoprotein (mmoL/L), *M* (SD)	2.1 (0.9)	2.2 (1.2)	.72^c^
High‐density lipoprotein (mmoL/L), *M* (SD)	1.17 (0.32)	1.07 (0.28)	.025^d^
eGFR, median (IQR)	90 (80, 90)	89 (66, 90)	.023^d^
Albumin to creatinine ratio—Median (IQR)	1.5 (0.5, 4.2)	2.0 (0.7, 12.1)	.023^d^
Smoking status, *n* (%)			.57^d^
Non‐smoker	49 (43%)	43 (39%)	
Ex‐smoker	46 (41%)	50 (45%)	
Smoker	18 (16%)	18 (16%)	

Abbreviations: BMI, body mass index; BP, blood pressure; eGFR, estimated glomerular filtration rate; IQR, interquartile range; SD, standard deviation.

^a^ Comparison of individual unadjusted covariates between AMOS and control groups.

^b^ Categorical measures using Fisher's exact test.

^c^ Estimated for continuous normally distributed measures using multiple linear regression (*t*‐test equivalent).

^d^ For measures with non‐normal distributions using ordered logistic regression (Mann–Whitney equivalent).

#### 3.1.2 Attendance

##### Intervention participants

Half of the participants attended all 10 core AMOS Clinic consultations (47%, *n* = 53). Overall, 12% (*n* = 14) attended 2–4 sessions, 13% (*n* = 15) 5–7 sessions and 74% (*n* = 84) 7–10 sessions. Forty‐two percent (*n* = 47) attended the 1.5‐h AMOS Group session, which did not apply to controls. Twenty percent attend the *Get The Most Out Of Life* and 5% the OPALL programs. Initially, 111 intervention participants attended the physiotherapist at recruitment, with two declining the assessment and program. However, 17% (*n* = 19) declined further sessions due to chronic pain following the initial assessment. Of the five potential physiotherapy consultations, 35% (*n* = 40) attended one, 55% (*n* = 62) two to three and only 11% (*n* = 12) four to five sessions: physiotherapy access was limited in the second year and was not offered. Over half (55%, *n* = 62) were referred to the psychologist, with 81% (*n* = 50) accepting and attending: 44% of the total intervention cohort.

##### Control participants

Overall, 11% (*n* = 12) of participants attended 2–4 usual Diabetes Centre appointment sessions, 22% (*n* = 24) 5–7 sessions and 72% (*n* = 80) 7–10 sessions. A third of control participants attended a public physiotherapist (32%, *n* = 36) and dietitian (32%, *n* = 35) throughout the AMOS trial and 15% (*n* = 17) a psychologist. Attendance varied from one to >10 consultations, with a mean of 3 visits across these allied health staff. A quarter of control participants (26%, *n* = 29) also had regular fortnightly contact with the CDE for insulin adjustments.

### 3.2 Primary outcomes

#### 3.2.1 Weight loss and BMI

With adjustment for age, sex, smoking and EOSS, intervention participants showed a greater change in mean body weight loss at 12 months compared with controls (mean = −5.9 kg) (see Table 2). Differences between the groups were sustained at all time‐points, with a difference in mean weight loss of −9.0 kg at 24‐month follow‐up.

**TABLE 2:** Comparison of changes in body weight, body mass index (BMI), metabolic markers and blood pressure within each trial group at follow‐up visits.

	Usual care (Control) (*N* = 111)		Comparison	*p*	AMOS Clinic (intervention) (*N* = 113)		Comparison	*p*	Between‐group difference^a^		
	Mean (SD)	Δ^b^	95% CI		Mean (SD)	Δ^b^	95% CI		Δ^b^	95% CI	*p*
Body weight (kg)^c^											
Visit^d^: 0 months	116.6 (23.0)	0.00			113.4 (22.9)	0.00					
6 months	116.0 (22.0)	−0.6	(−2.02, 0.87)	.78	108.6 (21.9)	−4.8	(−6.24, −3.36)	<.0001	−4.2	(−6.33, −2.12)	<.0001
12 months	115.1 (21.2)	−1.5	(−3.34, 0.35)	.16	106.0 (21.2)	−7.4	(−9.16, −5.59)	<.0001	−5.9	(−8.53, −3.23)	<.0001
18 months	114.4 (20.9)	−2.2	(−4.54, 0.16)	.078	104.7 (20.9)	−8.7	(−10.9, −6.39)	<.0001	−6.5	(−9.83, −3.10)	.0001
24 months	114.3 (20.9)	−2.3	(−5.23, 0.66)	.20	102.1 (20.9)	−11.3	(−14.1, −8.42)	<.0001	−9.0	(−13.2, −4.77)	<.0001
BMI (kg/m^2^)^c^											
Visit^d^: 0 months	41.0 (7.6)	0.00			39.5 (7.5)	0.00					
6 months	40.7 (7.2)	−0.30	(−0.78, 0.17)	.38	37.8 (7.2)	−1.77	(−2.24, −1.30)	<.0001	−1.5	(−2.16, −0.78)	<.0001
12 months	40.4 (7.0)	−0.58	(−1.20, 0.04)	.076	36.9 (7.0)	−2.61	(−3.20, −2.02)	<.0001	−2.0	(−2.92, −1.15)	<.0001
18 months	40.2 (6.9)	−0.79	(−1.58, 0.01)	.054	36.4 (6.9)	−3.13	(−3.89, −2.37)	<.0001	−2.4	(−3.48, −1.21)	<.0001
24 months	40.3 (6.9)	−0.66	(−1.65, 0.34)	.35	35.4 (6.9)	−4.16	(−5.12, −3.20)	<.0001	−3.5	(−4.93, −2.08)	<.0001
Haemoglobin A_1c_ (%)^c^											
Visit^d^: 0 months	8.2% (1.6%)	0.00%			8.4% (1.6%)	0.00%					
6 months	8.1% (1.5%)	−0.08%	(−0.36%, 0.19%)	.91	8.0% (1.5%)	−0.45%	(−0.73%, −0.18%)	.0002	−0.37%	(−0.77%, 0.03%)	.09
12 months	7.9% (1.4%)	−0.30%	(−0.60%, 0.00%)	.050	7.9% (1.4%)	−0.56%	(−0.85%, −0.26%)	<.0001	−0.26%	(−0.69%, 0.18%)	.49
18 months	8.0% (1.3%)	−0.15%	(−0.49%, 0.18%)	.69	7.7% (1.3%)	−0.72%	(−1.05%, −0.39%)	<.0001	−0.57%	(−1.05%, −0.09%)	.012
24 months	7.9% (1.4%)	−0.25%	(−0.64%, 0.13%)	.34	7.5% (1.4%)	−0.88%	(−1.25%, −0.51%)	<.0001	−0.63%	(−1.17%, −0.08%)	.016
Estimated glomerular filtration rate (eGFR, mL/min/1.73 m^2^)^e^, ^f^											
Visit^d^: 0 months	77.6 (16.9)	0.00			80.2 (16.9)	0.00					
6 months	76.5 (16.8)	−1.09	(−3.14, 0.95)	.55	80.3 (16.8)	0.18	(−1.87, 2.22)	1.00	1.27	(−1.71, 4.25)	.27
12 months	76.3 (16.8)	−1.31	(−3.48, 0.86)	.44	81.2 (16.8)	1.09	(−1.07, 3.25)	.60	2.40	(−0.74, 5.54)	.049
18 months	75.4 (17.1)	−2.20	(−4.54, 0.14)	.075	80.6 (17.1)	0.42	(−1.92, 2.77)	.999	2.62	(−0.79, 6.03)	.048
24 months	74.5 (17.5)	−3.10	(−5.72, −0.48)	.013	79.1 (17.5)	−1.07	(−3.67, 1.54)	.77	2.04	(−1.72, 5.79)	.16
Systolic blood pressure (mmHg)^c^											
Visit^d^: 0 months	131 (17.3)	0.0			133 (17.3)	0.0					
6 months	130 (16.7)	−1.3	(−5.7, 3.1)	.92	126 (16.7)	−6.8	(−11.2, −2.5)	.0003	−5.6	(−11.9, 0.8)	.12
12 months	130 (16.3)	−0.8	(−5.2, 3.7)	.99	128 (16.3)	−5.5	(−9.8, −1.1)	.0081	−4.7	(−11.1, 1.8)	.28
18 months	130 (16.0)	−1.3	(−5.8, 3.3)	.93	128 (16.1)	−5.7	(−10.2, −1.2)	.0062	−4.4	(−11.0, 2.1)	.35
24 months	132 (16.3)	1.3	(−3.4, 6.1)	.93	130 (16.2)	−3.5	(−8.1, 1.1)	.22	−4.8	(−11.6, 2.0)	.30
Diastolic blood pressure (mmHg)^c^											
Visit^d^: 0 months	73 (9.4)	0.0			74 (9.4)	0.0					
6 months	72 (9.4)	−1.0	(−3.5, 1.5)	.77	73 (9.4)	−1.2	(−3.6, 1.3)	.65	−0.2	(−3.7, 3.4)	1.00
12 months	71 (9.5)	−2.0	(−4.5, 0.5)	.16	73 (9.5)	−1.4	(−3.8, 1.1)	.52	0.6	(−2.9, 4.2)	.99
18 months	73 (9.6)	−0.3	(−2.8, 2.2)	1.00	73 (9.6)	−1.1	(−3.6, 1.3)	.68	−0.8	(−4.4, 2.8)	.982
24 months	74 (9.8)	0.3	(−2.2, 2.8)	1.00	74 (9.7)	−0.3	(−2.7, 2.2)	.998	−0.6	(−4.2, 3.0)	1.00
Total cholesterol (mmol/L)^c^											
Visit^d^: 0 months	4.2 (1.1)	0.00			4.3 (1.1)	0.00			0.08		
6 months	4.2 (1.1)	−0.01	(−0.22, 0.20)	1.00	4.1 (1.0)	−0.20	(−0.41, 0.01)	.070	−0.19	(−0.50, 0.12)	.44
12 months	4.1 (1.0)	−0.07	(−0.29, 0.14)	.87	4.1 (1.0)	−0.16	(−0.38, 0.05)	.21	−0.09	(−0.40, 0.22)	.96
18 months	4.1 (1.0)	−0.10	(−0.33, 0.12)	.67	4.1 (1.0)	−0.16	(−0.38, 0.06)	.27	−0.05	(−0.38, 0.27)	1.00
24 months	4.0 (1.0)	−0.13	(−0.37, 0.11)	.51	4.1 (1.0)	−0.18	(−0.41, 0.05)	.19	−0.05	(−0.39, 0.29)	1.00
LDL cholesterol (mmol/L)^c^											
Visit^d^: 0 months	2.2 (1.0)	0.00			2.1 (1.0)	0.00					
6 months	2.1 (0.9)	−0.11	(−0.30, 0.08)	.50	1.9 (0.9)	−0.22	(−0.41, −0.03)	.013	−0.12	(−0.39, 0.16)	.80
12 months	2.1 (0.9)	−0.15	(−0.35, 0.06)	.26	2.0 (0.9)	−0.17	(−0.37, 0.04)	.15	−0.02	(−0.31, 0.28)	1.00
18 months	2.0 (0.9)	−0.22	(−0.44, 0.01)	.064	2.0 (0.9)	−0.10	(−0.32, 0.12)	.70	0.12	(−0.21, 0.44)	.89
24 months	2.0 (0.9)	−0.17	(−0.42, 0.09)	.36	2.0 (0.9)	−0.11	(−0.36, 0.14)	.70	0.05	(−0.31, 0.42)	1.00
HDL cholesterol (mmol/L)^c^											
Visit^d^: 0 months	1.1 (0.4)	0.00			1.2 (0.4)	0.00					
6 months	1.1 (0.4)	0.05	(−0.02, 0.13)	.29	1.1 (0.4)	−0.01	(−0.09, 0.06)	.99	−0.07	(−0.18, 0.04)	.47
12 months	1.1 (0.4)	0.04	(−0.03, 0.12)	.49	1.2 (0.4)	0.01	(−0.07, 0.08)	1.00	−0.04	(−0.15, 0.07)	.91
18 months	1.2 (0.4)	0.09	(0.01, 0.16)	.03	1.2 (0.4)	0.03	(−0.05, 0.10)	.87	−0.06	(−0.17, 0.05)	.63
24 months	1.2 (0.4)	0.09	(0.01, 0.17)	.025	1.1 (0.4)	−0.01	(−0.09, 0.07)	1.00	−0.10	(−0.21, 0.02)	.14
Leptin (ng/mL)^c^											
Visit^d^: 0 months	53.6 (40.0)	0.0			49.6 (37.1)	0.0					
12 months	61.2 (41.6)	7.6	(−0.96, 16.2)	.17	46.7 (39.2)	−2.9	(−10.8, 5.02)	.88	−10.5	(−23.0, 1.96)	.20
24 months	83.9 (51.2)	30.3	(19.3, 41.3)	<.0001	52.7 (48.1)	3.2	(−6.80, 13.1)	.93	−27.1	(−42.7, −11.5)	.0002

Abbreviations: CI, confidence interval; eGFR, estimated glomerular filtration rate; HDL, high‐density lipoprotein; LDL, low‐density lipoprotein; SD, standard deviation.

^a^ The between‐group differences in the trial at each of the subsequent visits have been corrected for the difference at the initial visit.

^b^ Outcome mean (SD) and change (Δ; 95% CIs; *p*‐values corrected for multiple comparisons by the Sidak method) between visits estimated using mixed‐effects linear regression.

^c^ Adjusted for age, sex, smoking and EOSS.

^d^ Nominal visit time following initial measurement appointment and initiation of either AMOS Clinic or usual care.

^e^ Adjusted for age, sex, smoking, EOSS and HbA_1c_.

^f^ The rate of decline in eGFR in the usual care group was 1.30 mL/min/1.73 m^2^/year and in the AMOS clinic group 0.46 mL/min/1.73 m^2^/year: difference up to 18 months 1.76 mL/min/1.73 m^2^/year (95% CI −0.37, 3.90; *p* = 0.058).

BMI changes also favoured the intervention groups. At 6 months, the intervention group had a greater reduction in mean BMI (−1.5 kg/m^2^) compared with controls, with a larger change at 24 months, (−3.5 kg/m^2^). Cumulatively, more intervention participants underwent bariatric metabolic surgery during the trial (*n* = 10, 8.8% vs. *n* = 5, 4.5%) (see Table 3), although the numbers are small, hence conclusions uncertain. Sensitivity analyses excluding all bariatric surgery recipients during the trial and follow‐up showed larger relative differences in BMI for the intervention group at 6 months (−1.8 kg/m^2^) and 24 months (−3.9 kg/m^2^) compared with controls (see Table 3).

**TABLE 3:** Comparison of the change in body mass index (BMI) in usual care and AMOS Clinic groups at different follow‐up visits in those with or without previous bariatric surgery.

		Usual diabetes care (control)		Comparison			AMOS Clinic (intervention)^¶^		Comparison		
		*N* (%)	Mean (SD)	Δ^a^	95% CI	*p*	*N* (%)	Mean (SD)	Δ^a^	95% CI	*p*
Comparison of change from first visit to subsequent visits within‐group with or without bariatric previous surgery separately											
Participants who did not undergo bariatric surgery^b^											
Visit^c^	0 months	111	40.7 (8.7)	0.0			113	40.0 (7.1)	0.0		
	6 months	109	40.3 (8.5)	−0.14	(−0.52, 0.23)	.46	113	38.1 (7.1)	−1.80	(−2.17, −1.43)	<.0001
	12 months	108	40.0 (8.2)	−0.23	(−0.71, 0.24)	.34	108	37.2 (6.8)	−2.45	(−2.91, −1.99)	<.0001
	18 months	107	39.8 (8.3)	−0.35	(−0.96, 0.25)	.25	105	36.7 (6.4)	−2.88	(−3.46, −2.30)	<.0001
	24 months	106	39.9 (8.0)	−0.25	(−1.00, 0.50)	.52	103	35.7 (6.2)	−3.90	(−4.63, −3.18)	<.0001
Participants undergoing bariatric surgery during the trial period^b^											
Visit^c^, ^d^	6 months	2 (1.8%)	47.2 (14.4)				0	0			
	12 months	3 (2.7%)	47.5 (8.1)				5 (4.4%)	38.9 (6.9)			
	18 months	4 (3.6%)	43.9 (6.9)				8 (7.0%)	36.0 (7.7)			
	24 months	5 (4.5%)	44.3 (7.8)				10 (8.8%)	34.0 (6.1)			

Abbreviations: CI, confidence interval; SD, standard deviation.

^a^ Outcome mean (SD) and change (Δ; 95% CIs; *p*‐values corrected for multiple comparisons by the Holm method) between visits estimated using mixed‐effects linear regression.

^b^ Adjusted for age, sex, smoking, EOSS and HbA_1c_.

^c^ The comparison between AMOS Clinic (intervention) and usual diabetes care (control) groups at the first visit is the natural difference, while the differences at each of the subsequent visits have been corrected for the difference at the initial visit.

^d^ Nominal visit time following initial measurement appointment and initiation of either AMOS Clinic (intervention) or usual diabetes care (control).

##### Rates of 5% and 10% body weight loss

Participants in the intervention group achieved a faster clinically significant reduction in body weight than in the control group. Four times as many in the intervention group achieved a 5% reduction in BMI (38% vs. 9%; incidence rate ratio (IRR) 4.28; 95% CI 1.97, 9.29; *p* < .0001) at 6 months. By 24‐months, the deficit in the control group reduced (65% vs. 31%; IRR 2.10; 95% CI 1.46, 3.02; *p* < .0001). However, a 5‐fold difference in achieving a 10% reduction in BMI appeared at 12 months (25% vs. 4%; IRR 5.67; 95% CI 1.91, 16.9; *p* = .001), which persisted at the 2‐year visit (35% vs. 8%; IRR 5.61; 95% CI 2.40, 13.1; *p* < .0001).

#### 3.2.2 HbA_1c_

Within‐group findings showed intervention participants experienced a greater decrease in mean HbA_1c_ at 24 months compared with controls (−0.88% vs. −0.25%) (see Table 2). The difference between the groups in change of mean HbA_1c_ showed a greater reduction for the intervention group at 6 months (−0.37%). Similarly, differences between‐groups were sustained across the time‐points, with a difference in mean HbA_1c_ of −0.63% at 24‐month follow‐up.

### 3.3 Secondary outcomes

#### 3.3.1 Renal function

With adjustment for age, sex, smoking, EOSS and HbA1c, controls showed a greater change in mean eGFR decline at 12 and 18 months compared with intervention participants (2.40 and 2.62 mL/min/1.73 m^2^ respectively) (see Table 2). In all participants, the difference between eGFR change was a rate of 1.30 mL/min/1.73 m^2^/year in controls versus 0.46 mL/min/1.73 m^2^/year in the intervention group up to 18 months, an improvement difference of 1.76 mL/min/1.73 m^2^/year (see footnotes in Table 2). These results were exploratory due to sample size limitations, that is, a 50% minimum relative improvement would have required 1122 participants per group, assuming the effect size suggested in the current trial continued in the larger numbers. One control and three intervention participants had an eGFR <20 mL/min/1.73 m^2^, of whom one died of renal failure from each group.

#### 3.3.2 Blood pressure

Intervention participants showed a small decline in mean systolic BP improvement at 6 months (−6.8 mmHg), with the improvement diminishing over time but no change in controls (see Table 2). No differences were observed in diastolic BP at any time‐point. Between‐groups systolic BP differences could not be distinguished in this sample.

#### 3.3.3 Lipid profile

No mean total or LDL cholesterol differences were observed at any time‐point within and between groups (see Table 2). Over three‐quarters were on lipid‐lowering medication (see Table 1).

#### 3.3.4 Leptin

Intervention participants showed a greater difference in mean leptin level at 12 months (−10.5 ng/mL) compared with controls, which was larger at 24 months (−27.1 ng/mL) (see Table 2). The within‐group difference showed a marked increase in mean leptin level in controls by 24 months (30.3 ng/mL), whereas it remained stable in intervention participants.

#### 3.3.5 Liver function tests

The rate of decline in GGT was greater in the intervention than control group (difference in change in geometric mean GGT, −3.67 units/year [95% CI −6.08, −1.10; *p* = .0057]). There was no change in other LFTs in either group.

#### 3.3.6 Obesity staging

The proportion of intervention participants in Obesity Stages II (severe) and III (morbid) combined reduced from 70.8% (*n* = 80) at recruitment to 42.4% (*n* = 48) at 24 months (odds ratio [OR] 0.004; 95% CI 0.000, 1.42; *p* = .075), whereas no changes in proportions were seen in the control group (OR 0.26; 95% CI 0.002, 24.6; *p* = .96), a significant difference between treatments (OR 0.017; 95% CI 0.005, 0.054; *p* < .0001) (see Table 4).

**TABLE 4:** Proportion of participants with different grades of obesity in the AMOS Clinic and usual care groups.

Obesity grade		Episode				
		Initial visit, *N* (%)	6‐months, *N* (%)	12‐months, *N* (%)	18‐months, *N* (%)	24‐months, *N* (%)
AMOS Clinic (intervention)						
Overweight	(BMI <30 kg/m^2^)	0 (0.0%)	5 (4.4%)	10 (8.9%)	12 (10.6%)	15 (13.3%)
Obesity Stage I	(BMI ≥30–34.9 kg/m^2^)	33 (29.2%)	44 (38.9%)	41 (36.3%)	43 (38.1%)	50 (44.3%)
Obesity Stage II	(BMI ≥35–39.9 kg/m^2^)	34 (30.1%)	26 (23.0%)	30 (26.6%)	28 (24.8%)	24 (21.2%)
Obesity Stage III	(BMI ≥40 kg/m^2^)	46 (40.7%)	38 (33.6%)	32 (28.3%)	30 (26.6%)	24 (21.2%)
Total		113	113	113	113	113
Change from^a^ initial visit	OR *p*‐Value	1.00	0.10 (0.02, 0.64) .0078	0.037 (0.002, 1.42) .027	0.018 (0.000, 1.42) .084	0.004 (0.000, 1.42) .075
Rate of change per month		OR	0.33 (0.11, 1.01)	*p*‐Value .052		
Usual diabetes care (control)						
Overweight	(BMI <30 kg/m^2^)	0 (0.0%)	3 (2.7%)	5 (4.5%)	5 (4.5%)	5 (4.5%)
Obesity Stage I	(BMI ≥30–34.9 kg/m^2^)	32 (28.3%)	31 (27.9%)	27 (24.3%)	29 (26.1%)	26 (23.4%)
Obesity Stage II	(BMI ≥35–39.9 kg/m^2^)	30 (27.0%)	32 (28.8%)	31 (27.9%)	31 (27.9%)	36 (32.4%)
Obesity Stage III	(BMI ≥40 kg/m^2^)	49 (44.1%)	45 (40.5%)	48 (43.2%)	46 (41.4%)	44 (39.6%)
Total		111	111	111	111	111
Change from^a^ initial visit	OR *p*‐Value	1.00	0.44 (0.11, 1.74) .67	0.45 (0.039, 5.11) .95	0.28 (0.008, 9.31) .92	0.26 (0.002, 24.6) .96
Rate of change per month		OR	0.84 (0.27, 2.63)	*p*‐Value .99		
Comparison: OR^b^		0.26 (0.05, 1.32)	0.24 (0.079, 0.70)	0.082 (0.027, 0.30)	0.064 (0.021, 0.20)	0.017 (0.005, 0.054)
*p*‐Value		.42	.0041	<.0001	<.0001	<.0001
Rate of change		OR	0.39 (0.30, 0.51)	*p*‐Value <.0001		

Abbreviation: BMI, body mass index.

^a^ Comparison of obesity stage in each treatment group over 24 months.

^b^ Comparisons of differences in proportions of participants in different obesity stages between the treatment groups (AMOS Clinic minus Usual Diabetes Care). Comparison of distribution in AMOS Clinic and Usual care groups expressed as odds ratio (OR; 95% confidence intervals; *p*‐values) estimated using mixed‐effects ordered logistic regression, adjusted for age, sex, smoking and EOSS, and corrected for multiple comparisons by the Sidak method. An odds ratio of 1.00 indicates no difference, OR <1.00 indicates intervention patients had greater decline than controls.

#### 3.3.7 Glucose‐lowering medication

Insulin usage was unchanged in the control group (+1 from 102 units/day/year initially), while it declined in the intervention group (−17 from 100 units/day/year; mean difference −18 units/year; 95% CI −29, −6; *p* = .004). GLP‐1 use increased from 33 to 38 (30%–34%; *N* = 111; IRR per year 1.06, 95% CI 0.95, 1.20, *p* = .30) participants in control group, and 31 to 51 (27%–45%; *N* = 113; IRR per year 1.17, 95% CI 1.03, 1.33, *p* = .013) participants in intervention group, although the trial was too small to be certain that the difference was real (IRR per year 1.10, 95% CI 0.93, 1.31, *p* = .26).

## 4 DISCUSSION

### 4.1 Main findings

In this trial, AMOS Clinic participants saw greater weight, BMI and HbA_1c_ changes and less eGFR decline compared with controls. The results suggest an interdisciplinary obesity model delivering tailored, individually coordinated care for people with obesity and type 2 diabetes in rural settings can sustainably reduce weight and improve metabolic markers compared with usual diabetes care.

#### 4.1.1 Weight loss and BMI

AMOS intervention participants achieved weight loss comparable to participants in an Australian study of a multidisciplinary Metabolic Rehabilitation Program (MRP) for adults with diabetes and BMI > 30 kg/m^2^.^14^ Participants participated in a supervised, more intensive physical activity program, allied health integration and 6‐monthly consultations with an endocrinologist specialising in obesity; weight loss medications were not used. MRP study participants had similar characteristics to AMOS but were less likely to be in Obesity Stage III, potentially enabling more intensive physical activity because of lesser comorbidities and weight‐related limitations.

Studies with more intensive interventions, like the US‐based Look AHEAD trial, initially showed more pronounced weight loss compared with AMOS but lacked sustainability. Look AHEAD examined the effects of employing strict caloric restriction (1500–1800 kcal/day) and intense unsupervised exercise (>200 min/week) over 4 years, tapering support until year nine, compared with diabetes support and education on cardiovascular morbidity and mortality.^27^, ^28^ Despite achieving greater mean weight loss than AMOS at 12 months, Look AHEAD participants had a lower baseline mean BMI, suggesting fewer comorbidities affecting self‐care.^27^, ^28^ Regardless, the magnitude of these differences in weight and HbA_1c_ were not sustained at 24 months. Look AHEAD similarly used weight loss medication Orlistat when participants did not achieve a 10% body weight loss at 6 months; however, glucose‐lowering medication was adjusted according to rates of hypoglycaemic episodes only. By contrast, the AMOS trial focused on aligning insulin reductions with changes in caloric restrictions to reduce hunger and hypoglycaemia and aiding the transition to weight‐neutral/loss glucose‐lowering medications. Infrequently, low‐dose appetite suppressants (e.g. topiramate) were introduced after 12 months to support participants as new habits formed.

Short‐term success may be achieved through intensive exercise/calorie reduction; by contrast, the careful medication adjustment offered by AMOS, coupled with the development of self‐maintenance skills, resulted in longer‐term sustainable change. Prolonged caloric reduction poses challenges for most people with obesity because of the interplay of multiple metabolic pathways.^4^, ^29^, ^30^ In the AMOS trial, Saxenda (Liraglutide 3 mg), a GLP‐1 analogue, was used to support weight loss for those preparing for bariatric surgery, made available through a restricted government‐funded program. Additionally, participants used Orlistat as a tool to identify and reduce high‐fat foods, aiding self‐maintenance and long‐term success despite potential gastrointestinal effects.

Compared with a UK study examining a multidisciplinary obesity clinic focused on sustainable lifestyle changes, where a third of participants had diabetes (*n* = 73) and 67.4% classified as Obesity Stage III,^31^ AMOS Clinic participants were twice as likely to maintain >10% weight loss at 24 months (35% vs. 16%). The key difference between studies lies with all participants having diabetes and attending a tertiary Diabetes Centre enabling timely insulin and glucose‐lowering medication adjustments during clinic visits, aligned with reduced caloric or nutrition advice (e.g. low carbohydrate). This integrated approach likely facilitating weight loss while maintaining stable glucose levels and the patient's confidence. By contrast, the UK study may have experienced delays in titrating glucose‐lowering medication because participants were referred to their primary general practitioner, while bariatric surgery was managed by alternate bariatric services. These differences in care delivery may explain the varied weight loss outcomes between studies—highlighting the effectiveness of the AMOS Clinic's integrated interdisciplinary care, including shared‐care with bariatric services, and the emphasis on self‐maintenance skills, delivered in‐house.

#### 4.1.2 HbA_1c_ outcomes

In our study, interdisciplinary obesity care outperformed usual diabetes care in improving HbA_1c_, consistent with evidence showing weight loss improves metabolic markers.^12^, ^28^, ^32^, ^33^, ^34^ Like MRP, it took over 12‐months for AMOS intervention participants to see larger reductions in mean HbA_1c_.^14^ This highlights the time needed to manage obesity and adjust weight‐gaining, glucose‐lowering medicines without compromising diabetes control.^10^, ^14^, ^35^

AMOS showed a higher mean HbA1c in‐group difference than Look AHEAD intervention participants despite having higher mean BMI and HbA_1c_, reflecting their higher metabolic burden. While Look AHEAD trial participants experienced a similar decline in HbA_1c_ across groups at 12 months, this was not sustained at 24 months.^27^, ^28^ Timely adjustments to glucose‐lowering medicines, aligned with caloric reduction advice and interdisciplinary support, were critical in motivating AMOS Clinic participants. Our study and others^10^, ^14^, ^31^ suggest involving diverse health professionals who can manage glucose‐lowering medications within obesity teams results in better metabolic outcomes. By contrast, the UK study reported negligible in‐group differences; diabetes participants were excluded when deemed ‘poorly controlled’ or referred for separate diabetes management.^31^

The AMOS trial saw a near 1% reduction in mean HbA1c, an important target linked to reduced microvascular disease and cardiovascular morbidity and mortality.^25^ Unlike other studies, the AMOS Clinic prioritised cultivating long‐term self‐maintenance skills. This involved understanding triggers and motivators related to eating habits, weight fluctuation, stress and physical activity. By incorporating effective strategies into participants' lives to manage these factors, the approach resulted in a more sustained improvement in HbA1c.

### 4.2 Secondary outcomes findings

Weight loss positively contributed to reduced cardiovascular risk by improving several associated metabolic markers, including systolic BP and eGFR.

#### 4.2.1 Blood pressure outcomes

Our study observed a small reduction in systolic BP associated with weight loss, comparable with other studies,^15^, ^28^, ^31^ although the result is equivocal due to small sample size. The Look AHEAD study found clinically meaningful improvements in systolic BP with modest weight loss.^28^ Similarly, the MRP study reported improvements in systolic BP associated with improved BMI and fitness,^14^ although a Type 2 error was likely due to small numbers. Similar to the AMOS trial, their study did not evaluate adherence to lipid‐lowering and antihypertensive pharmacological agents; this may be an essential self‐maintenance strategy to review.

#### 4.2.2 Lipid levels outcomes

The AMOS trial suggests more attention is required for managing lipid profiles. While most participants were on lipid‐lowering medication at recruitment, there was more leniency about adjusting/starting lipid‐lowering medicines on the indication lipids may improve with dietary changes. However, delaying lipid‐lowering medication resulted in some participants having LDL and HDL cholesterols outside diabetes targets. In the MRP study, LDL and HDL cholesterols changed at the same rate in both groups^14^; however, intervention participants had a more improved LDL cholesterol, suggesting the intensity of their exercise program helped reduce LDL. The Look AHEAD trial demonstrated a strong association between improved HDL and fitness,^27^, ^28^ suggesting an exercise physiologist/physiotherapist's input to the AMOS Clinic may improve lipid levels through the metabolic effects of improved fitness.

#### 4.2.3 Leptin level outcomes

Our study found that despite exhibiting similar leptin resistance at recruitment, the control group, with less weight loss, experienced increased leptin levels. Leptin resistance hinders long‐term weight loss despite dietary improvements in ‘leptin resistance’ individuals.^4^, ^11^ Gruzdeva et al.'s review identified that as body fat mass reduces, leptin levels reduce, leading to hunger, increased appetite, reduced motivation to exercise and suppression of energy expenditure.^36^ We suggest ongoing support over 24 months, incorporating transition to weight‐neutral/loss diabetes medications that did not enhance appetite, was paramount for successful weight loss. This extended support helps people develop self‐maintenance strategies to manage competing metabolic pathways.

#### 4.2.4 Renal function outcomes

AMOS intervention participants experienced little eGFR decline, thus preserving renal function over the 2 years of the study, but in the longer term, the issue may be a significant concern. The control groups' rate of decline mirrored the control group in the Lipid‐lowering and Onset of Renal Disease (LORD) double‐blinded randomised trial in 132 subjects with mild–moderate chronic kidney disease in a similar Tasmania population.^37^ However, the statistical significance was borderline because of the small number of cases in the AMOS trial with eGFR<60 mL/min/1.73 m^2^ (LORD inclusion criteria). Overweight normotensive men following a mild caloric‐deficit low‐sodium diet, 1‐h aerobic exercise and a weekly 1‐h private counselling session demonstrated improved renal function at 11 months after similar weight loss to AMOS.^38^ Long‐term follow‐up in people with obesity revealed a seven‐fold increase in end‐stage renal disease relative risk, irrespective of hypertension or diabetes status,^39^ suggesting managing obesity is essential for protecting renal function. While this trial was not able to definitively confirm or refute an effect on rate of decline in renal function due to sample size issues, it would be important that any future studies include renal function as an outcome, aiming to permit meta‐analyses to combine trial data to resolve this issue in the future.

#### 4.2.5 Absolute CVD risk

This paper omitted these scores since they proved unhelpful in measuring change in cardiovascular risk for individuals with diabetes, consistently scoring >15%. Consequently, meaningful change was not discernible, aligning with other evidence.^40^

## 5 IMPLICATIONS FOR PRACTICE

### 5.1 Tailoring glucose‐lowering medications to weight loss efforts

AMOS findings suggest that reducing glucose‐lowering medicines, like insulin, along with caloric reduction, contributes to successful weight loss. In our earlier qualitative study, AMOS Clinic participants positively described the planned individually‐tailored approach to prescribing glucose‐lowering medications emphasising it supported ongoing self‐care and weight management.^41^ While insulin is commonly prescribed due to physician familiarity and the growing complexities of diabetes medications, it is essential to recognise it is one of many treatment options for managing type 2 diabetes, unlike type 1 diabetes.^3^, ^43^ Pivotally, Australia is facing an obesity epidemic, and as a growth hormone, it is crucial to acknowledge that insulin hinders weight loss efforts.^4^, ^18^, ^42^, ^43^ Studies have shown insulin therapy leads to weight gain and increases sedentary behaviour.^44^, ^45^ For instance, in the ‘Stepping Up Program’, where practice nurses helped with regular insulin titration, participants experienced weight gain as an adverse outcome.^45^ To address this, promoting Quality Use of Medicines skills can reduce a potential disproportionate focus on insulin and foster appropriate medication regimens.^29^, ^41^, ^43^

### 5.2 A dedicated, experienced team for managing obesity

AMOS aimed to foster self‐maintenance skills through an interdisciplinary health team offering obesity care. Prior research found that participants valued AMOS Clinic's individually‐tailored and multidisciplinary approach, facilitating personalised strategies for ongoing self‐care and weight management.^41^ Weight management can be challenging for medical physicians, who struggle with weight‐related conversations; however, people with diabetes are often prescribed weight‐gaining medications.^30^, ^46^ Additionally, health professionals may harbour implicit and explicit weight biases, which are yet to be understood.^4^, ^47^ Yet, 5%–10% body weight loss improves insulin sensitivity, endothelial function, and inflammation and coagulation markers, prevents type 2 diabetes progression and reduces cardiovascular risk, while 10%–15% clinically improves obstructive sleep apnoea, non‐alcoholic steatohepatitis, depression and mobility.^8^, ^9^, ^12^ The AMOS trial showed an interdisciplinary team skilled in managing obesity and diabetes collaborating with people living with both conditions can improve health outcomes.

### 5.3 Rural interdisciplinary obesity clinics

Understanding barriers and facilitators to self‐care is essential for improving the health of people with obesity. Promoting effective self‐care, including adequate sleep, physical activity, less sedentary behaviour, nutritious food, appropriate medical care and socialising, is linked to obesity reduction.^4^ Interdisciplinary clinics like AMOS are needed in rural areas to develop self‐maintenance skills to target these factors. Look AHEAD outcomes highlighted a strong, negative association between fitness and BMI, highlighting that people with obesity are less physically active than those less overweight.^27^, ^28^ Thus, there is a need to find an optimum way to enhance physical fitness in people already living with obesity. However, the Australian Government's National Obesity Strategy 2022–2032 prioritises promoting healthier options over healthcare strategies.^18^ While healthier options are essential for preventing obesity, delivering care to those already living with obesity and diabetes is paramount, especially in rural areas with higher obesity prevalence.^1^

### 5.4 Economics

The economic burden of diabetes is substantial, with direct diabetes healthcare expenditure AUD$2.7 billion in 2019.^48^ The annual total excess healthcare cost for people living with obesity compared with average‐weight people is 26%, and for those with obesity and diabetes, 46%.^49^ The AMOS trial showed that investing in diabetes services for obesity management could improve metabolic risk markers, lowering the risk of secondary complications. For example, AMOS intervention provided a reno‐protective benefit, crucial as diabetes is a leading cause of end‐stage renal disease. Dialysis, a common treatment for this condition, costs $125 000/person/year in Australia.^50^ Even a modest decrease in numbers requiring dialysis could fund the clinic, benefiting the healthcare system and individuals. While a complete economic evaluation of the AMOS Clinic model was beyond this trial's scope, such an investigation is warranted.

### 5.5 Limitations

Participant loss due to non‐attendance or lack of interest in the trial treatment was higher in the AMOS intervention group (75/292, 26% vs. 23/206, 11%; Fisher's exact *p* = .0001) than control. No difference due to other reasons occurred between groups. Randomisation occurred before recruitment for logistical reasons (see Figure 1). Also, due to a procedural misunderstanding, the last 29 participants were invited and assigned study numbers based on their order of presentation, deviating from the intended random allocation. The difference in baseline characteristics may result from random variation, a difference in recruitment behaviours given response rate, or represent a selection bias due to the study's unblinded nature, potentially indicating intervention participants were more committed to health improvement and treatment adherence. The differences may also represent a higher disease burden in the intervention group who required more glucose‐lowering medication to achieve the same mean HbA1c. Sensitivity analysis when assuming missing cases mirrored changes seen in the control group saw a small reduction in the benefit seen in BMI reduction in the intervention group.

The renal function analysis is exploratory due to sample size limitations. However, unexpected variability in eGFR levels between time‐points suggests a pattern of regression‐to‐mean. Also, routine laboratory analysis reported higher values of eGFR as ‘>90 mL/min/1.73 m^2^’, truncating the possible range of values. Further studies are needed to definitively determine the AMOS Clinic's effects on renal function, including full‐range eGFR reporting. Additionally, physiotherapy access was limited in the second 12 months, reducing treatment availability for some AMOS participants, potentially impacting weight loss outcomes.

## 6 CONCLUSIONS

This trial demonstrated that participants attending the AMOS Clinic model for managing people living with obesity and type 2 diabetes experienced greater clinically relevant improvements than those receiving usual diabetes care. The collaborative interdisciplinary care model focused on addressing the metabolic complexities of managing both conditions through coordinated, individually‐tailored interdisciplinary healthcare, medication review, diet and physical activity support, psychological care and appropriate bariatric surgery referral. Participants were drawn from a rural Australian region, suggesting that centralising coordinated obesity care in one treatment centre could feasibly be implemented in a rural setting, overcoming common geographical, economical and healthcare availability barriers to accessing care.

## AUTHOR CONTRIBUTIONS

*Conceptualization*: Giuliana O. Murfet. *Methodology*: Giuliana O. Murfet and Sharon P. Luccisano. *Software*: Giuliana O. Murfet. *Validation*: Giuliana O. Murfet, Iain K. Robertson, Sharon P. Luccisano and Michelle L. Kilpatrick. *Formal analysis*: Iain K. Robertson. *Secondary analysis and cross coding*: Michelle L. Kilpatrick, Giuliana O. Murfet and Sharon P. Luccisano. *Data curation*: Iain K. Robertson and Giuliana O. Murfet. *Writing—original draft preparation*: Giuliana O. Murfet and Michelle L. Kilpatrick. *Writing—review and editing*: Giuliana O. Murfet, Iain K. Robertson, Michelle L. Kilpatrick. and Sharon P. Luccisano. *Project administration*: Giuliana O. Murfet. *Funding acquisition*: Giuliana O. Murfet. All authors have read and agreed to the published version of the manuscript.

## FUNDING INFORMATION

The study was funded by a grant from the National Partnership Agreement—Tasmanian Assistance Package through the Department of Health and Human Services (Tasmania).

## CONFLICT OF INTEREST STATEMENT

No conflict of interest was declared.

## ETHICS STATEMENT

Ethics approval gained from the Tasmania Health and Medical Human Research Ethics Committee—Ethics approval number: H0014324.

## Supporting information


**Data S1.** Supporting information.

## Data Availability

The data used to support the findings of this study are available from the corresponding author upon request and in alignment with ethics.
