# Supplementary material for: Effect of interdisciplinary obesity care on metabolic markers and body weight in people with type 2 diabetes in a rural setting: A randomised controlled trial
Source: Clin Obes. 2024 Nov 7;15(2):e12715. doi: 10.1111/cob.12715 (PMC11907095; doi:10.1111/cob.12715)
Supplement: Supplementary file 1 — Data S1. Supporting information. [file COB-15-e12715-s001.pdf]

CONSORT CHECKLIST

Table. CONSORT 2010 Checklist of Information to Include When Reporting a Randomized Trial[a]

Section and Topic	Item No.	Checklist Item	Reported on Page No.
Title and abstract	1a	Identification as a randomized trial in the title	1 – line 4
	1b	Structured summary of trial design, methods, results, and conclusions (for specific guidance see CONSORT for abstracts)	1 – lines 17-39
Introduction<br>Background and objectives	2a	Scientific background and explanation of rationale	2 – lines 41-66
	2b	Specific objectives or hypotheses	2 – lines 66-72
Methods<br>Trial design	3a	Description of trial design (such as parallel, factorial) including allocation ratio	3 – lines 81-85
	3b	Important changes to methods after trial commencement (such as eligibility criteria), with reasons	NA
Participants	4a	Eligibility criteria for participants	3 – lines 78-80<br>Figure 2
	4b	Settings and locations where the data were collected	2 – lines 74-76
Interventions	5	The interventions for each group with sufficient details to allow replication, including how and when they were actually administered	3 & 4 – lines 98-131<br>Figure 1
Outcomes	6a	Completely defined prespecified primary and secondary outcome measures, including how and when they were assessed	4 – lines 132-151
	6b	Any changes to trial outcomes after the trial commenced, with reasons	NA
Sample size	7a	How sample size was determined	5 – lines 168-172
	7b	When applicable, explanation of any interim analyses and stopping guidelines	NA
Randomization<br>Sequence generation	8a	Method used to generate the random allocation sequence	3 – lines 86-88
	8b	Type of randomization; details of any restriction (such as blocking and block size)	3 – lines 86-96
Allocation concealment mechanism	9	Mechanism used to implement the random allocation sequence (such as sequentially numbered containers), describing any steps taken to conceal the sequence until interventions were assigned	3 – lines 88-91
Implementation	10	Who generated the random allocation sequence, who enrolled participants, and who assigned participants to interventions	3 – lines 91-96
Blinding	11a	If done, who was blinded after assignment to interventions (for example, participants, care providers, those assessing outcomes) and how	NA
	11b	If relevant, description of the similarity of interventions	NA
Statistical methods	12a	Statistical methods used to compare groups for primary and secondary outcomes	4 – lines 152 - 167
	12b	Methods for additional analyses, such as subgroup analyses and adjusted analyses	NA
Results<br>Participant flow (a diagram is strongly recommended)	13a	For each group, the numbers of participants who were randomly assigned, received intended treatment, and were analyzed for the primary outcome	Figure 2
	13b	For each group, losses and exclusions after randomization, together with reasons	Figure 2
Recruitment	14a	Dates defining the periods of recruitment and follow-up	5 – lines 174-182

	14b	Why the trial ended or was stopped	NA, stopped as planned
Baseline data	15	A table showing baseline demographic and clinical characteristics for each group	Table 1
Numbers analyzed	16	For each group, number of participants (denominator) included in each analysis and whether the analysis was by original assigned groups	5 – lines 175-182<br><br>Tables 2, 3 & 4
Outcomes and estimation	17a	For each primary and secondary outcome, results for each group, and the estimated effect size and its precision (such as 95% confidence interval)	6 to 7 – lines 207 to 264<br><br>Tables 2, 3 & 4
	17b	For binary outcomes, presentation of both absolute and relative effect sizes is recommended	Tables 1, 2, 3 & 4
Ancillary analyses	18	Results of any other analyses performed, including subgroup analyses and adjusted analyses, distinguishing prespecified from exploratory	NA Table 3
Harms	19	All important harms or unintended effects in each group (for specific guidance see CONSORT for harms)	None to report
Comment<br>Limitations	20	Trial limitations, addressing sources of potential bias, imprecision, and, if relevant, multiplicity of analyses	12 – lines 461-478
Generalizability	21	Generalizability (external validity, applicability) of the trial findings	7 to 10 – lines 266 to 406
Interpretation	22	Interpretation consistent with results, balancing benefits and harms, and considering other relevant evidence	7 to 10 – lines 266 to 406
Other information<br>Registration	23	Registration number and name of trial registry	3 – line 85<br><br>14 – Reference 24 line 546
Protocol	24	Where the full trial protocol can be accessed, if available	3 – line 85<br><br>14 – Reference 24 line 546
Funding	25	Sources of funding and other support (such as supply of drugs), role of funders	0 – Tile page with acknowledgements

[a] We strongly recommend reading this statement in conjunction with the CONSORT 2010 Explanation and Elaboration for important clarifications on all the items. If relevant, we also recommend reading CONSORT extensions for cluster randomized trials, noninferiority and equivalence trials, nonpharmacological treatments, herbal interventions, and pragmatic trials. Additional extensions are forthcoming: for those and for up-to-date references relevant to this checklist, see http://www.consort-statement.org.

©2010 American Medical Association. All rights reserved.
